# Infections Due to Multidrug-Resistant Bacteria in Oncological Patients: Insights from a Five-Year Epidemiological and Clinical Analysis

**DOI:** 10.3390/microorganisms7090277

**Published:** 2019-08-21

**Authors:** Eleni Isidora A. Perdikouri, Kostoula Arvaniti, Dimitrios Lathyris, Fani Apostolidou Kiouti, Eleni Siskou, Anna Bettina Haidich, Christos Papandreou

**Affiliations:** 1 Department of Medical Oncology, Aristotle University of Thessaloniki School of Medicine, 54124 Thessaloniki and General Hospital of Volos, 38222 Volos, Greece; 2 Intensive Care Unit, Infection Control Unit, Antibiotic Stewardship Committee, Papageorgiou University affiliated Hospital, 56403 Thessaloniki, Greece; 3 Intensive Care Unit, G. Gennimatas Hospital, 54635 Thessaloniki, Greece; 4 Department of Hygiene and Biostatistics, Aristotle University of Thessaloniki School of Medicine, 54124 Thessaloniki, Greece; 5 Microbiology and Biochemistry Department, Papageorgiou University affiliated Hospital, 56403 Thessaloniki, Greece; 6 Department of Medical Oncology, Aristotle University of Thessaloniki School of Medicine, 54124 Thessaloniki, Greece

**Keywords:** cancer, multidrug resistance, epidemiology, risk factors

## Abstract

Bacterial infections are frequent complications in cancer patients. Among them, those caused by multidrug-resistant (MDR) bacteria increase morbidity and mortality mainly because of limited therapeutic options. Current knowledge regarding MDR infections in patients with solid tumors is limited. We assessed the epidemiology and risk factors of increased mortality in these patients. In this retrospective five-year single cohort observational study, we included all oncological patients with MDR infections. Cancer-related parameters, comorbidities, prior use of antibiotics, previous surgical interventions and hospitalization, as well as the use of invasive procedures were investigated as potential risk factors causing adverse outcomes. Seventy-three patients with MDR infection were included: 37% with carbapenem-resistant *Klebsiella pneumoniae*, 24% with oxacillin-resistant *Staphylococcus aureus* (MRSA) and 21% with carbapenem-resistant *Acinetobacter baumanni*. Previous colonization with MDR bacteria was detected in 14% patients, while 20% of the patients presented MDR colonization or infection at ward admission. Mortality during the infection episode was 32%. Duration of hospitalization and CRP were statistically significant risk factors of mortality, whereas administration of guided antibiotics was a protective factor. Knowledge of local epidemiology of MDR bacteria can help physicians promptly identify cancer patients at risk of MDR infections and initiate timely effective empirical antibiotic treatment that can eventually improve the overall therapeutic management.

## 1. Introduction

Bacterial infection is one of the most frequent complications in immunosuppressed cancer patients, concerning both solid and hematological malignancies. Nowadays, the extensive spread of multidrug-resistant (MDR) bacteria among humans, animals and environmental reservoirs has created novel unanticipated epidemiological patterns of those bacteria in healthcare facilities [1]. Without exception, cancer patients, frequently cared for and hospitalized in healthcare structures, are also affected by MDR infections often associated with considerable morbidity, mortality and financial burden [2,3]. Changes in global epidemiology of infections in oncological patients have occurred overtime, characterized by a shift from prevalent Gram-negative bacteria between the 1960s and 1970s to Gram-positive ones ten years later and, beyond this, a new restitution of Gram-negative bacteria in the last 20 years in many countries [4,5,6]. Among Gram-negative bacteria, Enterobacteriaceae, mainly *Klebsiella pneumoniae*, *Pseudomonas aeruginosa* and *Acinetobacter baumannii* resistant to carbapenems, have been increasingly implicated as infecting pathogens in cancer patients, with variable frequency depending on the reporting country and continent [7,8,9,10]. Additionally, most existing guidelines propose antibiotic schemes for neutropenic or septic oncological patients, whereas empirical antibiotic regimens for suspected MDR infection in non-neutropenic and non-septic cancer patients are not clearly defined [11,12,13]. On the other hand, inadequate antibiotic treatment, either empirical or guided by antibiotic susceptibility tests, exposes patients to increased risk of adverse outcome, especially in neutropenic bacteremic patients suffering from MDR infections [2,8,10,14,15,16].

In the era of MDR spread within the entire healthcare setting, data regarding MDR infections in cancer patients—not necessarily neutropenic or septic—are limited. We conducted the present five-year retrospective study to describe the current epidemiology of MDR infections and identify outcomes and risk factors associated with mortality in a cohort of patients with solid tumors.

## 2. Materials and Methods

### 2.1. Study Characteristics

This retrospective observational single-center cohort five-year study was conducted in the 25-bed Department of Medical Oncology in Papageorgiou Hospital, Thessaloniki, Greece. The study included patients from 1 January 2013 to 31 December 2017. All cancer patients aged 18 years and older who presented an infection due to MDR bacteria during the study period were included in the study. In case of recurrence of infection after the initial hospital discharge, only the initial episode was analyzed.

### 2.2. Data Collection

Data recordings included demographic data (age and gender), comorbidities (diabetes mellitus, thrombotic events such as stroke or coronary heart disease, heart and renal failure, obesity, hypertension), oncological history (primary location of the disease, existence of metastatic lesions and lines of chemotherapies), surgical intervention during the last 6 months, recent chemotherapy (during the last 15 days) or radiotherapy during the last 30 days, use and category of antibiotics during the last 6 months, hospitalization in the Oncology Department or in other departments of the hospital or other hospitals in the last 6 months, existence of central venous catheter (port-a-cath or PICC (peripherically inserted central catheter), stents, pigtails, drains and stomias, microbiological samples and pathogen(s) isolated (identification and antibiotic susceptibility tests), existence of fever and its duration, the sepsis status at ward admission according to Sepsis-3 criteria [17], laboratory parameters (the worst values before MDR infection diagnosis) such as hematological (white blood cells, neutropenia) [18] and biochemical ones (urea, creatinine, CRP-C-reactive protein, total proteins and albumin), infection site imaging, colonization with MDR bacteria, empirical antibiotics administered for the analyzed infection episode and their duration, effectiveness of antibiotic treatment, use of guided (or targeted) by antibiotic susceptibility tests antibiotics and their duration, days of hospitalization and outcome of the analyzed infection episode, discharge or death and if death was related to the current infection episode according to the referring physician.

### 2.3. Ethics

The study was conducted in accordance with the Declaration of Helsinki and was approved on 21 December 2018 by the Institutional Review Board (IRB) of the hospital (Number of IRB Decision 307), whereas written informed consent was obtained from all patients alive at the time of the study (after verbal communication and information by phone call). For all other cases (i.e., dead patients), the Institutional Review Board of the hospital waived the need for patient consent, since this was a retrospective non-interventional study that reported only aggregated fully-anonymized data, and no financial conflict of interest for the hospital, patients’ insurance or the researchers existed. All data collected during this study were kept confidential.

### 2.4. Study Outcomes

We aimed to describe the clinical characteristics of all patients with MDR infections during the five-year study period and to present mortality with its associated predictors.

### 2.5. Definitions

Patients were considered to have an MDR infection if they had at least one positive for MDR pathogen clinical sample, and if clinical, laboratory and/or imaging data indicated infection and/or if diagnosis of infection was recorded in the patient’s records. Bacteria were considered MDR if they were resistant (or with intermediate susceptibility) to at least three of the following antibiotic classes: piperacillin/tazobactam, cephalosporins (ceftriaxone, ceftazidime, cefepime), carbapenems (imipenem/meropenem), monobactams, aminoglycosides (gentamicin, amikacin) and/or fluoroquinolones, regarding Enterobacteriacae, *Klebsiella pneumoniae*, *Acinetobacter baumannii* and *Pseudomonas aeruginosa*, and if they were resistant to vancomycin for *Enterococcus faecium*, and resistant to oxacillin for *Staphylococcus aureus* (MRSA) [19].

Colonization with multidrug-resistant bacteria was defined as at least one culture of nasal swab positive for MRSA or at least one culture of rectal swab positive for the rest of the MDR bacteria as defined above. Swabs were collected for colonization screening and not for the diagnosis of infection. Colonization cultures were usually collected at ward admission (within the first 48 h) in order to detect admitted MDR-colonized patients according to the Hospital Infection Control Unit guideline or later at any time during hospitalization for the studied episode.

Microbial identification was performed using commercially available panels (Vitek II, Biomerieux, Paris, France) and standard biochemical and/or enzymatic tests. Antibiotic susceptibility was tested using the microdilution method following the Clinical Laboratory Standard Institute (CLSI) guidelines [20].

Fever was considered if oral temperature ≥38.3 °C (101 °F) or was above 38.0 °C (100.4 °F) for at least 1 h. Neutropenia was defined as an absolute neutrophil count ≤500 neutrophils/μL.

Empirical antibiotic regimen was regarded in case of antibiotic treatment before microbiology results were available and communicated to physicians. Antibiotic regimens were considered guided if they were prescribed after the notification of the antibiotic susceptibility tests. Effective empirical antibiotic treatment was defined as the regimen including at least one antibiotic with in vitro activity against the isolated MDR pathogen, according to the antibiotic susceptibility tests.

Mortality as study outcome was defined as death within the hospital stay for the studied infection episode.

### 2.6. Statistical Analysis

Data were analyzed using the R studio 1.1.442 program. Considering descriptive statistics, mean and standard deviation were used for quantitative continuous variables that were parametric (with normal distribution, checked by Shapiro test, boxplots and histograms), whereas median and interquartile ranges were used for non-parametric continuous variables. We analyzed risk factors associated with mortality using univariate and multivariate logistic regression analyses. In the univariate analysis, odds ratio (OR) and 95% confidence interval (CI) were calculated. A *p*-value of < 0.05 was considered statistically significant. Variables with low *p*-values, as well as those with clinical significance indicated by current scientific reports despite their higher calculated *p*-value, were analyzed in multivariate analysis. Logistic regression model was used for multivariate analysis.

## 3. Results

### 3.1. Demographical Data, Comorbidities and Cancer-Related Data

Seventy three oncological patients suffered from infections due to multidrug-resistant bacteria during the study period.

Patients’ clinical characteristics are described in Table 1. Among the patients, 39 (53%) were males and 34 (47%) females. Diabetes mellitus, smoking, thrombosis, hypertension and heart and renal failure were found in 13 (18%), 37 (51%), 3 (5%), 42 (58%), 1 (1%) and 19 (26%) patients, respectively. The most frequent diagnoses were bladder cancer in 10 (14%) patients, lung cancer in 9 (13%), colon cancer in 11 (15%), uterine carcinoma in 8 (11%) and breast cancer in 8 (10%) patients. Among the patients, 71 (97%) had metastatic disease, 12 (17%) had received radiotherapy during the last 30 days, 18 (26%) had recent surgical intervention in the last 6 months and 28 (38%) had received chemotherapy in the last 2 weeks before the patients’ admission in the Oncology department. Only 3 out of 73 patients had never received chemotherapy; the great majority of the patients had a performance status greater than 2. Twenty five (34%) patients had a central venous catheter (either a port-a-cath or a PICC), 13 (18%) had a stent and 23 (32%) a stomia.

### 3.2. Infection-Related Data

Thirty seven out of 73 (51%) patients had fever during the recorded episode and 10 (14%) presented neutropenia. Most of the patients (50, 69%) had received antibiotics and 55 (74%) had prior hospitalization in the last 6 months. Seventeen (23%) patients were considered septic upon admission. Mean duration of hospitalization for the infection episode was 17.4 days (range 1–63). Death was recorded in 23 (32%) patients, in 22 of them it was considered as infection-related (Table 2). Death occurred in 10.52 days (mean, range 0–32) since MDR infection diagnosis.

### 3.3. Microbiological Results

Multidrug-resistant bacteria were recovered as follows: 27 (37%) patients had infection due to MDR *K. pneumoniae,* 17 (24%) due to MRSA, 15 (21%) due to MDR *A. baumannii*, 4 (5%) due to MDR *P. aeruginosa*, 3 (4%) due to MDR *E. coli* and 2 (3%) due to vancomycin-resistant *E. faecium*. In 5 (6%) patients, the implicated MDR bacteria were more than two (Table 2).

Twenty-one (29%) patients had bacteremia, 10 (14%) had positive sputum culture, 27 (37%) had positive urine culture, 4 (5%) had positive trauma fluid culture, 6 (8%) had positive ascites culture, 4 (6%) had central venous catheter tip positive culture and 1 (1%) had positive pleural fluid culture (Table 2). For 16 (22%) patients, the referring physician’s infection diagnosis was based not only on clinical and microbiological results, but also on imaging; computed tomography was the most common form of imaging (10 patients, 14%).

Ten (14%) patients were colonized with MDR bacteria prior to infection diagnosis and 15 (20%) were colonized or infected with MDR pathogens at the time of ward admission (admitted cases). Fifty (69%) patients had received empirical antibiotic treatment, which was effective in 10 (13%) patients. Thirty-three (47%) patients had received antibiotics, guided (and considered effective) by antibiotic susceptibility tests.

### 3.4. Univariate and Multivariate Analysis

In univariate analysis, white blood cells, C-reactive protein (CRP) and days of hospitalization for the analyzed episode increased the risk of death (OR:0.999, *p* 0.003; OR: 0.940, *p* 0.020; OR:0.956, *p* 0.023, respectively), while sepsis and guided antibiotics were protective factors (OR:0.022, *p* < 0.001 and OR: 0.225, *p* 0.006, respectively). Neither cancer-related parameters, nor comorbidities, interventions and procedures or previous hospitalization reached statistical significance (Table 3).

Because of the low number of events in the studied cohort (23 deaths), we tested multiple models in the multivariate analysis. In every model, two variables were analyzed (Table S1). CRP and duration of hospitalization were significantly associated with adverse outcome (OR: 0.933, 95%CI: 0.877–0.983, *p* = 0.016; OR: 0.959, 95%CI: 0.919–0.996, *p* = 0.036, respectively). Administration of antibiotics according to antibiotic susceptibility tests was found to significantly increase the probability of survival (OR: 0.198, 95% CI: 0.062–0.579, *p* = 0.004). Effective empirical antibiotics showed a trend for a protective effect, without, however, statistical significance (OR: 0.281, 95%CI: 0.068–0.956, *p* = 0.056) (Table 4).

## 4. Discussion

We aimed to define current epidemiology of MDR infections in cancer patients and to identify risk factors of adverse outcomes in the era of growing evidence for hospital-wide spread of these bacteria in many countries and continents. This is one of the few studies concerning MDR infections in cancer patients, not obligatory neutropenic or septic. We showed that the difficulties in treatment of MDR infections and the easy spread of those bacteria concern not only typical patient populations, such as critically ill patients in Intensive Care Unit (ICU) settings, but, also, oncological patients that, until recently, were considered a patient population of little concern for MDR infection.

In our cohort, multidrug resistant *K. pneumoniae*, *A. baumannii* and to a lesser extent *P. aeruginosa*, as well as MRSA, caused infections with considerable mortality and, most importantly, did so in patients without neutropenia or sepsis, which represents an alarming issue. Guided and, by definition, effective antibiotics significantly decreased mortality, while length of hospital stay and CRP were independent risk factors of adverse outcome.

Multidrug resistant *A. baumannii* and *K. pneumoniae* were the most prevalent pathogens in the present cohort of cancer patients, as is the case in other hospital settings and countries [1,2,3,10,21,22]. MRSA represented an important part of the infection episodes, while vancomycin-resistant *E. faecium* was rarely isolated, similarly to other reports with a predominance of MDR Gram-negative bacteria over MRSA or VRE in the general patient population or in immunocompromised patients [6,8,23,24].

A considerable fatality rate of 32% was observed among the analyzed patients and death was infection-related in 30% of cases. Marin et al. reported a comparable fatality rate of 32% in a cohort of 489 cancer patients with bacteremia, with the majority of the cases being non-neutropenic [8]. According to their results, MDR bacteria were isolated in 13% of the patients and 39.2% overall 30-day mortality was recorded, higher than the entire cohort (32%) [8]. In 2015, Freire et al. presented 83 infections due to carbapenem-resistant *K.pneumoniae* in patients with solid malignancies (13% with neutropenia); mortality reached 57.8% for all infections and exceeded 70% for bacteremia cases [2]. In a case-control study including both solid and hematological malignancies, Fukuta et al. presented fatality rates up to 41.9% for cancer patients with MDR *A. baumannii* infections [3].

Factors associated with increased fatality rates in our patients were CRP and duration of hospitalization, while guided antibiotics during hospitalization for the infection episode decreased mortality. Effective empirical antibiotics presented a trend for statistical significance without reaching it, possibly due to the limited number of analyzed events (23 fatal cases). Variables related to comorbidities, cancer stage and relevant therapies, interventions, catheters or sepsis stage were not risk factors of increased mortality. Contrasting our results, Marin et al. identified cancer- and sepsis-related factors as independent risk factors for the overall case-fatality rate in patients with solid tumors and bacteremia [25]. A protective effect of appropriate antibiotics against carbapenem-resistant *K. pneumoniae* infections in cancer patients (88% without neutropenia) was shown in the study by Freire et al., along with an increased fatality rate significantly associated with sequential organ failure assessment (SOFA) score, along with the need for ICU stay after the diagnosis of infection and acute kidney injury [2]. Similarly to our cohort, ineffective antibiotics against MDR *A. baumannii* bacteremia in cancer patients significantly increased the likelihood of death occurrence in a retrospective analysis of 95 patients, along with carbapenem resistance, septic shock and pneumonia as the source of bacteremia [15].

CRP was significantly associated with fatal outcomes in our cases, a finding that has not been reported previously to the best of our knowledge. CRP is commonly considered as a prognostic marker of severity related to cancer stage, severity of infection or both. In contrast to our results, Lee et al. found that in cancer patients admitted to the Emergency Department with febrile neutropenia, CRP, among other potential predictors (lower blood pressure, platelet count, quick sequential (sepsis-related) organ failure assessment (qSOFA) score) failed to accurately predict ICU admission or in-hospital mortality [26].

In the present study, duration of hospitalization increased the risk of fatal events, possibly reflecting the severity of the infection episode or the prolonged exposure of the patient to healthcare-associated risks, such as MDR bacteria acquisition, invasive procedures or drug-adverse events, as is the case in other healthcare-associated infections (ventilator-associated pneumonia, central catheter-related infection, urinary tract infection, etc.). This finding needs further evaluation before conclusions are reached.

Empirical antibiotic treatment was initiated in 69% of the patients, although only 14% and 23% presented neutropenia or sepsis, respectively, which is in accordance with existing guidelines [11,13]. Forty-seven percent of the patients received guided, and so, effective antibiotics nevertheless, only 13% of the patients received effective empirical antibiotic regimens. One possible explanation is that guidelines and proposed algorithms for empirical treatment at the time the study patients were hospitalized did not take into consideration MDR infections in non-neutropenic, non-septic patients (as was the majority of our cohort) [11]. Similarly to our findings, Marin et al. reported inadequate empirical antibiotic therapy for 23% of cancer bacteremic patients, with a higher proportion in those with MDR strains (69%) [8].

Fourteen percent of our patients had previous MDR colonization, which could have possibly increased the risk of MDR infection. In a recent systematic review and meta-analysis, Alevizakos et al. reported high colonization rates with extended-spectrum β-lactamase-producing Enterobacteriaceae (ESBL-PE) among patients with solid or haematological malignancy (one in five patients with cancer was colonized with ESBL-PE), and this increases the risk of bacteremia with the same pathogen and created important reservoirs for horizontal spread between oncological hospitalized patients [27].

One of the strengths of the study is the important number of risk factors investigated. We searched meticulously among demographical data and comorbidities, cancer-related conditions, invasive procedures, infection-related variables and antibiotic treatment parameters (empirical and guided, effective and non-effective) to identify variables potentially influencing patients’ outcomes. The study represents one of the few research works on MDR infections in oncological patients and, to our knowledge, the first one in the Greek territory. Furthermore, the retrospective design allowed us to accurately estimate the diagnosis of infection and choose appropriately the eligible cases, as well as, to search widely over an extended period for the most relevant to MDR infection parameters, without interfering with treatment parameters.

The study has several limitations: it was a single-center retrospective data collection and the sample size was not adequately powered to detect all potential risk factors of mortality. Moreover, the impact of the kinetics of CRP on patient’s outcome was not explored. Because of the retrospective design of the study, we did not have CRP follow-up values at predefined time-intervals for all patients (only as prescribed by referring physicians), as such, the real influence of this parameter on outcome could not be accurately estimated.

## 5. Conclusions

Contemporary microbial patterns in oncological patients have evolved towards multidrug resistance. Infections due to MDR bacteria can negatively affect patients’ outcome.

Taken for granted the current threat of multidrug resistance, risk assessment for MDR infection is imperative for every cancer patient, and, if suspicion exists, prompt initiation of effective antibiotics seems to be a beneficial approach for the patient’s outcome.

There is an unmet need for surveillance of MDR bacteria, for effective antimicrobial regimens combined with antibiotic stewardship programs and for wide implementation of infection control measures in Oncology departments.

## Figures and Tables

**Table 1:** Patients’ clinical characteristics.

Clinical Characteristics	N (73)	%
Sex		
Male	39	53
Female	34	47
Comorbidities	54	74
Diabetes mellitus	13	18
Smoking	37	51
Thrombosis	3	5
Hypertension	42	58
Heart failure	1	1
Renal failure	19	26
Diagnosis (primarycancer)		
Bladder	10	14
Breast	8	10
Glioblastoma	2	3
Lung	9	13
Head and neck	6	9
Pancreas	3	4
Cholangiocarcinoma	2	3
Gastric	3	4
Colon	11	15
Prostate	1	1
Ovarian	3	4
Uterus	8	11
Cervical	5	7
Skin	2	2
Metastatic disease	71	97
Lines of chemotherapy		
First diagnosis—no chemotherapy	3	4
1st line	38	52
2nd line	22	30
3rd line	6	8
>4th line	4	6
Recent radiotherapy (last 1month)	12	17
Recent surgery	18	26
Intervention performed		
Biliary stent	1	3
Cystectomy	4	5
TAH/BSO	3	4
Stomia	3	5
Enterectomy	3	4
Orthopaedic surgery	2	3
Craniotomy	2	3
Others	1	1
Immunotherapy	1	1
Recent chemotherapy	28	38
Prior antibiotics (last 6months)	50	69
Prior hospitalization (last 6months)	55	74
Oncological ward	17	23
Other ward of the hospital	26	35
Other hospital	12	16
Central venous catheter	25	34
Port-a-cath	23	30
PICC	2	4
Stents	13	18
Biliary	5	6
Pigtails	8	12
Stomia	23	32
Bowel	1	2
Kidney	16	22
Trachea	3	4

TAH, total abdominal hysterectomy; BSO, bilateral-salpingo-oophorectomy; PICC, peripherically inserted central catheter.

**Table 2:** Infection-related characteristics.

Infection-Related Characteristics	N (73)	%
Patients	73	100
Sample type		
Blood culture	21	29
Sputum	10	14
Urine	27	37
Trauma	4	5
Ascites	6	8
CVC (port-a-cath or PICC)	4	6
Pleural	1	1
Implicated pathogens		
MDR *E. coli*	3	4
MDR *Pseudomonas aeruginosa*	4	5
MDR *Klebsiella pneumoniae*	27	37
MDR *Acinetobacter baumannii*	15	21
Vancomycin-resistant *Enterococcus faecium*	2	3
Oxacillin-resistant *Staphylococcus aureus* (MRSA)	17	24
>2 MDR bacteria implicated	5	6
Infection site imaging	16	22
X-ray	2	3
CT	10	14
MRI	4	5
MDR colonization prior to MDR infection	10	14
MDR admitted from other hospital	15	20
Fever	37	51
Neutropenia	10	14
Sepsis	17	23
Empirical antibiotics administered	50	69
Effective empirical antibiotics administered	10	13
Guided antibiotics administered	34	47
Outcome of the studied infection episode		
Dead	23	32
Alive	50	68
Death was infection-related	22	30

CVC, central venous catheter; PICC, peripherically inserted central catheter; MDR, multidrug-resistant; CT, computed tomography; MRI, magnetic resonance imaging.

**Table 3:** Univariate analysis of patients’ and infection-related characteristics.

Variables	OR	LCI (95%)	UCI (95%)	*p*-Value
Age	1009	0.958	1062	0.718
Sex				
Male	1000			
Female	0.929	0.343	2522	0.884
Comorbidities:	1005	0.308	3022	0.994
DM	0.312	0.088	1070	0.063
Smoking	0.710	0.258	1911	0.499
Thrombosis	1251	0.221	20,255	0.816
Obesity	0.790	0.237	2875	0.707
Hypertension	1062	0.386	2880	0.905
Renal failure	2036	0.632	7920	0.260
Lines of chemotherapy	0.917	0.576	1499	0.715
Radiotherapy	2623	0.618	18,112	0.239
Surgery	3137	0.904	14,685	0.097
Chemotherapy before	2226	0.780	7034	0.148
Days of chemotherapy	1304	0.951	2417	0.319
Previous use of antibiotics	1653	0.574	4686	0.344
Previous hospitalization	2571	0.862	7734	0.089
CVC (Port-a-cath or PICC)	1286	0.455	3877	0.642
Stents	0.686	0.199	2532	0.553
Stomia	0.605	0.213	1740	0.344
Fever	0.419	0.145	1144	0.096
Duration of fever	0.777	0.496	1205	0.253
Neutropenia	2000	0.451	14,039	0.406
Urea	1008	0.996	1024	0.248
Creatinine	1714	1032	3982	0.106
WBCs	0.999	0.999	0.999	0.003
Total proteins	1531	0.895	2743	0.131
Albumin	1140	0.453	2930	0.780
CRP	0.940	0.890	0.988	0.020
Imaging of infection site	0.357	0.112	1128	0.077
Sepsis	0.022	0.003	0.096	<0.001
MDR colonization	2000	0.451	14,038	0.406
Empirical antibiotics	0.343	0.089	1081	0.086
Effective empirical antibiotics	0.909	0.216	4646	0.899
Duration of empirical antibiotics	0.961	0.891	1034	0.289
Guided antibiotics	0.225	0.073	0.632	0.006
Duration of guided antibiotics	0.966	0.924	1005	0.103
Days of hospitalization	0.956	0.917	0.992	0.023

OR, odds ratio; DM, diabetes mellitus; CVC, central venous catheter; PICC, peripherically inserted central catheter; WBCs, white blood cells; CRP, c-reactive protein.

**Table 4:** Models with statistically significant results in univariate and multivariate analysis.

	Univariate Model				Multivariate Model			
Models	OR	LCI (95%)	UCI (95%)	*p*-Value	OR	LCI (95%)	UCI (95%)	*p*-Value
Model 1								
Neutropenia	2.000	0.451	14.039	0.406	1.755	0.337	13.412	0.531
CRP	0.940	0.890	0.988	0.020	0.943	0.892	0.991	0.026
Model 2								
Creatinine	1.714	1.032	3.982	0.106	2.074	0.947	5.982	0.118
CRP	0.940	0.890	0.988	0.020	0.933	0.877	0.983	0.016
Model 3								
Guided antibiotics	0.225	0.073	0.632	0.006	0.203	0.049	0.779	0.021
Duration of guided antibiotics	0.966	0.924	1.005	0.103	1.006	0.954	1.065	0.813
Model 4								
Empirical antibiotics	0.343	0.089	1.081	0.086	0.281	0.068	0.956	0.056
Guided antibiotics	0.225	0.073	0.632	0.006	0.198	0.062	0.579	0.004
Model 5								
CRP	0.940	0.890	0.988	0.020	0.935	0.878	0.987	0.021
Guidedantibiotics	0.225	0.073	0.632	0.006	0.217	0.058	0.709	0.015
Model 6								
Duration of empirical antibiotics	0.961	0.891	1.034	0.289	0.984	0.909	1.066	0.690
Days of hospitalization	0.956	0.917	0.992	0.023	0.959	0.919	0.996	0.036
Model 7								
Guided antibiotics	0.225	0.073	0.632	0.006	0.207	0.064	0.600	0.005
Effective empirical antibiotics	0.909	0.216	4.646	0.899	0.560	0.111	3.164	0.484

## Supplementary Materials

The following are available online at https://www.mdpi.com/2076-2607/7/9/277/s1.
