# Supplementary material for: Infections Due to Multidrug-Resistant Bacteria in Oncological Patients: Insights from a Five-Year Epidemiological and Clinical Analysis
Source: Microorganisms. 2019 Aug 21;7(9):277. doi: 10.3390/microorganisms7090277 (PMC6780124; doi:10.3390/microorganisms7090277)
Supplement: Supplementary file 1 [file microorganisms-07-00277-s001.pdf]

microorganisms

MDPI

Supplementary file

Table S1. Models tested for statistical significance in univariate and multivariate analysis.

Model		Univariate model				Multivariate model			
Model 1		OR	LCI(95%)	UCI(95%)	p-value	OR	LCI (95%)	UCI (95%)	p-value
Age		1.009	0.958	1.062	0.718	1.008	0.956	1.064	0.739
Sex	Male	1.000				0.974	0.347	2.767	0,961
	Female	0.929	0.343	2.522					
Model 2					0.884				
Age		1.009	0.958	1.062	0.718	1.009	0.958	1.064	0.712
Comorbidities		1.005	0.308	3.022	0.994	0.955	0.284	2.968	0.939
Model 3									
Age		1.009	0.958	1.062	0.718	1.013	0.960	1.068	0.626
DM		0.312	0.088	1.070	0.063	0.304	0.085	1.051	0.059
Model 4									
Age		1.009	0.958	1.062	0.718	1.012	0.959	1.064	0.687
Renal failure		2.036	0.632	7.920	0.260	2.055	0.637	8.012	0.254
Model 5									
Age		1.009	0.958	1.062	0.718	1.009	0.958	1.062	0.735
Lines of chemotherapy		0.917	0.576	1.499	0.715	0.922	0.578	1.510	0.733
Model 6									
Radiotherapy		2.623	0.618	18.112	0.239	2.267	0.509	15.943	0.327
Sex		3.137	0.904	14.685	0.097	2.897	0.820	13.684	0.126
Model 7									
Radiotherapy		2.623	0.618	18.112	0.239	2.826	0.652	19.790	0.211
Chemotherapy before		2.226	0.780	7.034	0.148	2.334	0.808	7.476	0.130
Model 8									
Sex		3.137	0.904	14.685	0.097	3.106	0.881	14.699	0.104
Chemotherapy before		2.226	0.780	7.034	0.148	2.203	0.756	7.086	0.161
Model 9									
Sex		3.137	0.904	14.685	0.097	2.679	0.744	12.767	0.161
Hospitalization		2.571	0.862	7.734	0.089	2.186	0.712	6.725	0.169
Model 10									
Radiotherapy		2.623	0.618	18.112	0.239	3.042	0.685	21.854	0.188
Hospitalization		2.571	0.862	7.734	0.089	2.819	0.923	8.849	0.069
Model 11									

Hospitalization		2.571	0.862	7.734	0.089	2.793	0.912	8.778	0.072
Chemotherapy before		2.226	0.780	7.034	0.148	2.428	0.826	7.994	0.121
Model 12									
Antibiotics before		1.653	0.574	4.686	0.344	1.015	0.264	3.526	0.982
Hospitalization		2.571	0.862	7.734	0.089	2.550	0.693	9.909	0.160
Model 13									
Antibiotics before		1.653	0.574	4.686	0.344	1.908	0.643	5.714	0.241
Chemotherapy before		2.226	0.780	7.034	0.148	2.465	0.843	8.066	0.113
Model 14									
Fever		0.419	0.145	1.144	0.096	0.399	0.136	1.101	0.08
Neutropenia		2.000	0.451	14.039	0.406	2.263	0.494	16.286	0.34
Model 15									
Chemotherapy before		2.226	0.780	7.034	0.148	2.059	0.681	6.925	0.216
Neutropenia		2.000	0.451	14.039	0.406	1.418	0.277	10.610	0.694
Model 16									
Neutropenia		2.000	0.451	14.039	0.406	1.755	0.337	13.412	0.531
CRP		0.940	0.890	0.988	0.020	0.943	0.892	0.991	0.026
Model 17									
Neutropenia		2.000	0.451	14.039	0.406	2.209	0.487	15.721	0.348
Creatinine		1.714	1.032	3.982	0.106	1.726	1.043	4.013	0.100
Model 18									
Creatinine		1.714	1.032	3.982	0.106	2.074	0.947	5.982	0.118
CRP		0.940	0.890	0.988	0.020	0.933	0.877	0.983	0.016
Model 19									
Hospitalization		2.571	0.862	7.734	0.089	2.443	0.809	7.422	0.111
MDR colonization		2.000	0.451	14.038	0.406	1.690	0.367	12.079	0.537
Model 20									
Hospitalization		2.571	0.862	7.734	0.089	2.961	0.948	9.628	0.063
CVC		1.286	0.455	3.877	0.642	1.683	0.560	5.616	0.370
Model 21									
CVC		1.286	0.455	3.877	0.642	1.365	0.478	4.163	0.569
MDR colonization		2.000	0.451	14.038	0.406	2.107	0.469	14.925	0.375
Model 22									

Antibiotics before		1.653	0.574	4.686	0.344	1.563	0.531	4.531	0.410
Empirical antibiotics		0.343	0.089	1.081	0.086	0.353	0.091	1.119	0.096
Model 23									
Antibiotics before		1.653	0.574	4.686	0.344	1.652	0.574	4.683	0.345
Effective empirical		0.909	0.216	4.646	0.899	0.918	0.216	4.732	0.910
Model 24									
Empirical antibiotics		0.343	0.089	1.081	0.086	0.375	0.089	1.318	0.145
Empirical treatment duration		0.961	0.891	1.034	0.289	0.986	0.908	1.074	0.737
Model 25									
Antibiotics guided		0.225	0.073	0.632	0.006	0.203	0.049	0.779	0.021
Duration of guided antibiotics		0.966	0.924	1.005	0.103	1.006	0.954	1.065	0.813
Model 26									
Empirical antibiotics		0.343	0.089	1.081	0.086	0.281	0.068	0.956	0.056
Antibiotics guided		0.225	0.073	0.632	0.006	0.198	0.062	0.579	0.004
Model 27									
Empirical treatment duration		0.961	0.891	1.034	0.289	0.952	0.881	1.028	0.210
Duration of guided antibiotics		0.966	0.924	1.005	0.103	0.963	0.921	1.003	0.079
Model 28									
Sex		3.137	0.904	14.685	0.097	2.952	0.832	13.992	0.121
Fever		0.419	0.145	1.144	0.096	0.443	0.151	1.234	0.125
Model 29									
Chemotherapy before		2.226	0.780	7.034	0.148	2.427	0.829	7.894	0.119
Empirical antibiotics		0.343	0.089	1.081	0.086	0.318	0.081	1.022	0.071

Model 30								
CRP		0.940	0.890	0.988	0.020	0.935	0.878	0.987	0.021
Antibiotics guided		0.225	0.073	0.632	0.006	0.217	0.058	0.709	0.015
Model 31									
Duration of guided antibiotics		0.966	0.924	1.005	0.103	1.027	0.950	1.113	0.497
Days of hospitalization		0.956	0.917	0.992	0.023	0.937	0.868	1.004	0.074
Model 32									
Fever		0.419	0.145	1.144	0.096	0.397	0.091	1.680	0.209
Duration of fever		0.777	0.496	1.205	0.253	1.034	0.551	2.047	0.918
Model 32									
Antibiotics guided		0.225	0.073	0.632	0.006	0.312	0.083	1.114	0.075
Days of hospitalization		0.956	0.917	0.992	0.023	0.980	0.934	1.025	0.386
Model 33									
Empirical treatment duration		0.961	0.891	1.034	0.289	0.984	0.909	1.066	0.690
Days of hospitalization		0.956	0.917	0.992	0.023	0.959	0.919	0.996	0.036
Model 34									
Antibiotics guided		0.225	0.073	0.632	0.006	0.207	0.064	0.600	0.005
Efffective empirical		0.909	0.216	4.646	0.899	0.560	0.111	3.164	0.484

CVC, Central Venous Catheter

MDR, Multidrug Resistant
